# Anxiety prevalence and its association with physical activity in patients with non-communicable diseases during COVID-19 lockdown: a cross-sectional study in Shanghai, China

**DOI:** 10.1186/s12889-022-14369-1

**Published:** 2023-02-13

**Authors:** Yanyun Li, Tianzhichao Hou, Minna Cheng, Ya Miao, Yeerzati Yeerjang, Chang-sheng Sheng, Kun Xue, Cui Wu, Sheng Zhang, Qinghua Yan, Jianfeng Pei, Qinping Yang, Jingyan Tian, Wanghong Xu, Yan Shi

**Affiliations:** 1 Shanghai Municipal Center for Disease Control and Prevention, Shanghai, China; 2 Department of Endocrine and Metabolic Diseases, Shanghai Institute of Endocrine and Metabolic Diseases, Ruijin Hospital, Shanghai Jiao Tong University School of Medicine, Shanghai, China; 3 Key Lab of Health Technology Assessment (National Health Commission), School of Public Health, Fudan University, Shanghai, China; 4 School of Public Health, Fudan University, Shanghai, China; 5 Center for Epidemiological Studies and Clinical Trials and Center for Vascular Evaluation, Shanghai Institute of Hypertension, Ruijin Hospital, Shanghai Jiao Tong University School of Medicine, Shanghai, China; 6 Center for Disease Control and Prevention of Baoshan District, Shanghai, China; 7 Shanghai National Clinical Research Center for Metabolic Diseases, Key Laboratory for Endocrine and Metabolic Diseases of the National Health Commission of the PR China, Shanghai Key Laboratory for Endocrine Tumor, State Key Laboratory of Medical GenomicsRuijin HospitalShanghai Jiao Tong University School of Medicine, Shanghai, 200025 China; 8 National Clinical Research Center for Aging and Medicine, Shanghai, China

**Keywords:** SARS-CoV-2, Quarantine, Diabetes mellitus, Hypertension, Anxiety disorders, Physical activity

## Abstract

**Background:**

Quarantine due to the COVID-19 pandemic may have created great psychological stress among vulnerable populations. We aimed to investigate the prevalence of anxiety and explore the association between physical activities (PA) and anxiety risk in people with non-communicable diseases during the period of COVID-19 lockdown.

**Methods:**

We conducted a cross-sectional telephone survey from February 25 to April 20, 2020, the period of COVID-19 lockdown in Shanghai. Up to 8000 patients with type 2 diabetes and/or hypertension were selected using multi-stage cluster random sampling. PA level was measured based on the International Physical Activity Questionnaire using Metabolic Equivalent for Task scores, while symptoms of anxiety were assessed by the 7-item Generalized Anxiety Disorder scale. Multiple logistic regression analyses were performed to evaluate the associations of type and level of PA with the risk of anxiety.

**Results:**

Of a total 4877 eligible patients, 2602 (53.4%) reported with anxiety, and 2463 (50.5%), 123 (2.5%) and 16 (0.3%) reported with mild, moderate, and severe anxiety. The prevalence of anxiety was higher in the females, the elders, non-smokers, non-drinkers, and patients with diabetes, and the associations of anxiety with sex, age, smoking, drinking and diagnosis of diabetes were significant. A significant negative association was observed for housework activities (OR 0.53, 95%CI: [0.45, 0.63], *p* < 0.001) and trip activities (OR 0.55, 95%CI: [0.48, 0.63], *p* < 0.001) with anxiety, but no significant was found for exercise activities (OR 1.06, 95%CI: [0.94, 1.20], *p* = 0.321). Compared with patients with a low PA level, those with a moderate (OR 0.53, 95%CI: [0.44, 0.64], *p* < 0.001) or a high PA level (OR 0.51, 95%CI: [0.43, 0.51], *p* < 0.001) had a lower prevalence of anxiety.

**Conclusion:**

This study demonstrates a higher prevalence of anxiety in patients with hypertension, diabetes, or both during the COVID-19 lockdown. The negative associations of housework and trip activities with anxiety highlight the potential benefit of PA among patients with non-communicable diseases.

**Supplementary Information:**

The online version contains supplementary material available at 10.1186/s12889-022-14369-1.

## Background

COVID-19 has become a severe threat to global health, causing terror and anxiety around the globe [1]. Some local governments adopted strict lockdown policies to control the pandemic of the disease and control the contagion effectively [2]. However,, the national restriction policies changed the normal lifestyle of people, leading to psychological problems and negative emotions such as escalating loneliness and social isolation in the victims [3, 4]. The prevalence of anxiety was reported to be 11.6% to 31.9% during the lockdown period in China [5–7], and even worse in patients with existing chronic diseases [8].

Previous studies have suggested that patients with chronic disorders were highly associated with worse psychological status, lower cognitive function, and deteriorating mental health [9, 10]. This could be partially attributed to mutually interaction with sleeping disorders, behavior problems, physical malfunction, and even heavy economic burden of treatment [10–13].

This association might be intensified during the pandemic and restriction of COVID-19 [14]. Lack of physical exercise, altered sleep quality and behavior, long-term sedentarism, shortage of medications, high medical expenditure, and severe mortality are considered possible mediators that contribute to mental health deterioration among people diagnosed with non-communicable diseases during the COVID-19 pandemic [15–19]. This implied that the specific population may suffer more psychological stress than usual. Given the large number of patients with non-communicable diseases in China, it is urgent to address the mental problems in these people during COVID-19 lockdown.

Physical activities (PA), such as mild walking, moderate cycling, and vigorous exercise, could cause excess energy expenditure. Previous studies have explored the associations between PA and health in multiple populations [20–22], and found that PA played an essential role in managing depression and anxiety [23]. Due to the limitation of outdoor activities, home-based PA was highly recommended during the COVID-19 pandemic [24]. Nevertheless, the previous results on the benefits of PA were controversial, probably due to diversity of the study populations [25, 26].

Therefore, in this study we investigate the prevalence of anxiety in Chinese patients with hypertension and / or type 2 diabetes, two common non-communicable diseases, during COVID-19 lockdown. We hypothesized that more PA would help improve the risk and symptoms of anxiety in the patients, and quantitatively evaluated the relationship between levels of PA and anxiety. Our results may provide strong evidence for clinical guidelines to assist patients with non-communicable diseases in dealing with psychological problems, particularly in the context of the ongoing pandemic of COVID-19.

## Methods

### Study design and population selection

**Fig. 1:**
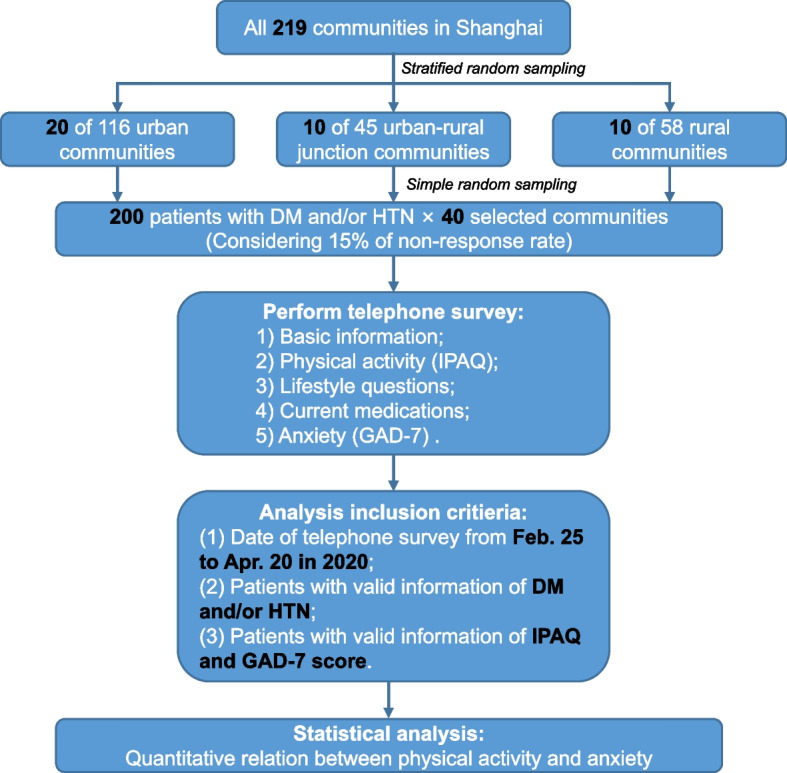
Flowchart of the study. Abbreviation: DM: Diabetes mellitus; HTN: Hypertension

A cross-sectional telephone survey was conducted by well-trained investigators while the lockdown/stay-home order was strictly imposed in Shanghai during the first half of 2020. A multi-stage random sampling was conducted in patients with type 2 diabetes and/or hypertension registered in the Shanghai Standardized Chronic Disease Management System. First, we randomly selected a total of 40 communities from urban (20 of 116 communities), urban–rural junction (10 of 45 communities), and rural areas (10 of 58 communities) of Shanghai. Then, we excluded the patients with missing telephone number or telephone number in wrong format and randomly selected 200 patients from all registered patients with diabetes and/or hypertension and with available telephone number in each selected patient. A total of 8,000 survey participants were selected (Fig. 1 and Supplement Note 1).

The need for Informed Consent was waived by the Ethics Committee of the Institutional Review Board of the Fudan University School of Public Health (IRB00002408 & FWA00002399) due to the nature of the study.

### Measures and questionnaire variables

This 15-min telephone survey comprised five major sections (Supplement Note). It included: 1) demographic information of participants (gender, age, weight, height, history of type 2 diabetes and hypertension); 2) PA level measured using the Chinese version of International Physical Activity Questionnaire (IPAQ); 3) lifestyle factors (smoking, drinking, and intake of meat, fish, vegetables, fruits and soy products); 4) current medications and medical treatment (kinds of medications and dose, self-report blood glucose and blood pressure); 5) Anxiety symptoms assessed using the 7-item Generalized Anxiety Disorder (GAD-7) scale.

All patients whose questionnaires met the following criteria were included in the analysis: (1) the date of recorded telephone survey was from February 25 to April 20 in 2020; (2) patients provided clear information on diagnosis of hypertension and/or diabetes; (3) patients provided valid information about IPAQ and GAD-7 score.

Age and gender were extracted from the questionnaires. BMI was calculated based on self-reported body weight and height in kg/m^2^. Smoking status was divided into three response options: current smokers, ex-smokers, and non-smokers. Drinking status was also divided into three groups: current drinkers, ex-drinkers, and non-drinkers. PA was collected based on the Chinese IPAQ questionnaire, including exercise activities, housework activities, and trip activities (biking and walking) [27]. The IPAQ evaluated the intensity and duration of PA in the past seven days. The levels of intensity included walking (mild), moderate-intensity PA, and vigorous-intensity PA. We used two scoring systems of IPAQ, short and long scales, to calculate the Metabolic Equivalent for Task (MET) score. The short-scale included three types of PA for MET calculation: 3.3 min/week for walking and mild PA, 4 min/week for biking and moderate PA, and 8 min/week for intensive PA. All participants were categorized into three groups according to the METs: low PA level (< 600 MET min/week); moderate PA level (≥ 600 and < 3000 MET min/week) and high PA level (≥ 3000 MET min/week). The validity and reliability of the Chinese version of IPAQ have been evaluated in previous studies [28, 29].

Anxiety disorder in patients during the recent COVID-19 pandemic and lockdown were assessed using the GAD-7 scale, which included the frequency that people were bothered by seven anxiety symptoms [30, 31]. Each symptom had 0 to 3 points. The Cronbach’s alpha of the GAD-7 was found to be 0.91 [32]. The total score of 0–4 were considered no/minimal anxiety, 5–9 as mild anxiety, 10–14 moderate anxiety, 15–21 severe anxiety. To ensure adequate cases, a total score over 5 indicated “with anxiety” in our study.

### Statistical analysis

Data were analyzed using the R software version 4.1.2. Basic statistical analyses of the main characteristics of interest, including means, standard deviations, frequencies, percentage, etc., were computed by sex of the patients. Categorical variables were described as counts with percentages; continuous variables were described as the median with interquartile range (IQR). We used the χ^2^ test and rank-sum test to compare differences between subgroups.

Multiple logistic regression analyses were performed to estimate odds ratios (OR) and 95% confidence interval [CI] of PA level with anxiety after adjusting for sex and age (model 1), additionally for BMI, smoking, and alcohol drinking (model 2), and additionally for preexisting diseases (model 3). We also used a restricted cubic spline (RCS) within logistic regression model to evaluate the potential non-linear associations between PA intensity and the risk of anxiety by using the median of 3360 min/week METs as the reference. All tests were two sided, and *P* < 0.05 after Benjamini–Hochberg correction of false discovery rate (FDR) was considered significant.

## Results

### Participant characteristics

A total of 4985 participants from 8000 calls answered the survey (62.3%), in which there were 4877 subjects whose questionnaires fully met our criteria (61.0%). The reason for the 3015 loss of follow-up was due to refusal (1019, 12.7%), incorrect phone number (1533, 19.2%), absence (450, 5.6%), and death (13, 0.2%). A total of 4821 subjects had complete data for all variables.

The basic characteristics of the 4877 participants are shown in Table 1. The median age of the participants was 61.5 years, and 2410 (49.4%) were female. 1187 (24.3%) had diabetes only, 2420 (49.6%) had hypertension only, and 1270 (26.0%) had both. The median BMI was 24.7 kg/m^2^. Most participants were non-smokers (71.9%) and non-drinkers (83.7%). 80.1% of participants had housework activities, with a median housework time of 1.5 h per day. Less than half of the participants had physical exercise (41.0%), with a median time of 2 h per day. A few participants (30.2%) had trip activity (biking and/or walking). The median MET was 3360 min/week, 4053 min/week for females, and 2373 min/week for males. The housework activity and MET were significantly higher in females than in males.

**Table 1:** Baseline characteristics of the study population

Characteristic	Total subjects (*n* = 4877)	Female (*n* = 2410, 49.4%)	Male (*n* = 2467, 50.6%)	*P*-value
Age (years)	61.5 (51.8, 70.9)	61.6 (52.3, 70.9)	61.5 (52.5, 70.9)	0.509
< 50	1052 (21.6)	504 (20.9)	548 (22.2)	
50–59	1177 (24.1)	588 (24.4)	589 (23.9)	
60–69	1295 (26.6)	658 (27.3)	637 (25.8)	
≥ 70	1353 (27.7)	660 (27.4)	693 (28.1)	
BMI (kg/m^2^)	24.7 (22.7, 26.8)	24.4 (22.3, 26.7)	24.8 (23.0, 27.0)	< 0.001*
< 18.5	92 (1.9)	54 (2.2)	38 (1.5)	
18.5–24.9	2483 (50.9)	1260 (52.3)	1223 (49.6)	
25.0–29.9	1763 (236.1)	810 (33.6)	953 (38.6)	
≥ 30	344 (7.7)	185 (7.7)	159 (6.4)	
Type of disease (%)				0.038
Diabetes only	1187 (24.3)	556 (23.1)	631 (25.6)	
Hypertension only	2420 (49.6)	1238 (51.4)	1182 (47.9)	
Both	1270 (26.0)	616 (25.6)	654 (26.5)	
Smoking (%)				< 0.001*
Current smoker	1062 (21.8)	24 (1.0)	1038 (42.1)	
Ex-smoker	308 (6.3)	5 (0.2)	303 (12.3)	
Non-smoker	3501 (71.9)	2378 (98.8)	1123 (45.6)	
Alcohol drinking (%)				< 0.001*
Current drinker	663 (13.6)	19 (0.8)	644 (26.2)	
Ex-drinker	129 (2.7)	8 (0.3)	121 (5.0)	
Non-drinker	4070 (83.7)	2380 (98.9)	1690 (68.8)	
Physical activity (%)				
Housework	3938 (80.1)	2142 (89.0)	1796 (72.9)	< 0.001*
Exercise	1998 (41.0)	955 (39.6)	1043 (42.3)	0.067
Trip activity	1461 (30.2)	710 (29.5)	751 (30.4)	0.512
MET (min/week)	3360 (1413, 5040)	4053 (2373, 5040)	2373 (792, 3954)	< 0.001*

Data presented as the number and percentage for categorical variables and the median with quartile for continuous variables

^*^Significant *p* value after FDR correction

### Prevalence of anxiety

The prevalence of anxiety in participants by characteristics was showed in Table 2. Up to 2602 (53.4%) subjects reported with anxiety, including 2463 (50.5%) with mild anxiety, 123 (2.5%) with moderate anxiety, and 16 (0.3%) with severe anxiety. A significant difference was found in prevalence of anxiety by smoking status, alcohol drinking status. The prevalence of anxiety was 57.4% in non-smokers, significantly higher than 45.9% in current smokers and 34.4% in ex-smokers (*p* < 0.001). Non-drinkers also had a higher prevalence than current drinkers and ex-drinkers (55.8% vs. 43.7% vs. 31.0%, *p* < 0.001). The prevalence of anxiety in patients with diabetes only and those with diabetes and hypertension were 55.0% and 55.2%, respectively, higher than that in patients with hypertension only (51.6%). The prevalence of anxiety did not significantly differ by sex, age group, BMI groups and history of non-communicable diseases in our subjects.

**Table 2:** Prevalence of mild to severe anxiety symptom in participants

	**Presence of anxiety**		***P*-value**	**Levels of anxiety**		
	**Without** **(score of 0–4)**	**With** **(score of 5–21)**		**Mild** **(score of 5–9)**	**Moderate** **(score of 10–14)**	**Severe** **(score of 15–21)**
No. participants (%)	2275 (46.6)	2602 (53.4)		2463 (50.5)	123 (2.5)	16 (0.3)
Sex (%)			*0.309*			
Female	1106 (45.9)	1304 (54.1)		1219 (50.6)	75 (3.1)	10 (0.4)
Male	1169 (47.4)	1298 (52.6)		1244 (50.4)	48 (1.9)	6 (0.2)
Age groups (%)			*0.828*			
< 50	497 (47.2)	555 (52.8)		533 (50.7)	18 (1.7)	4 (0.4)
50–59	558 (47.4)	619 (52.6)		590 (50.1)	26 (2.2)	3 (0.3)
60–69	601 (46.4)	694 (53.6)		661 (51.0)	30 (2.3)	3 (0.2)
≥ 70	619 (45.8)	734 (54.2)		679 (50.2)	49 (3.6)	6 (0.4)
BMI groups (kg/m^2^)			*0.885*			
< 18.5	47 (51.1)	45 (48.9)		39 (42.4)	5 (5.4)	1 (1.1)
18.5–24.9	1186 (47.8)	1297 (52.2)		1229 (49.5)	63 (2.5)	5 (0.2)
25.0–29.9	858 (48.7)	905 (51.3)		859 (48.7)	40 (2.3)	6 (0.3)
≥ 30.0	166 (48.3)	178 (51.7)		170 (49.4)	6 (1.7)	2 (0.6)
History of disease (%)			*0.046*			
Diabetes only	534 (45.0)	653 (55.0)		614 (51.7)	38 (3.2)	1 (0.1)
Hypertension only	1172 (48.4)	1248 (51.6)		1182 (48.8)	55 (2.3)	11 (0.5)
Both	569 (44.8)	701 (55.2)		667 (52.5)	30 (2.4)	4 (0.3)
Smoking (%)			< 0.001*			
Current smoker	575 (54.1)	487 (45.9)		472 (44.4)	13 (1.2)	2 (0.2)
Ex-smoker	202 (65.6)	106 (34.4)		99 (32.1)	6 (1.9)	1 (0.3)
Non-smoker	1492 (42.6)	2009 (57.4)		1892 (54.0)	104 (3.0)	13 (0.4)
Alcohol drinking (%)			< 0.001*			
Current drinker	373 (56.3)	290 (43.7)		283 (42.7)	6 (0.9)	1 (0.2)
Ex-drinker	89 (69.0)	40 (31.0)		37 (28.7)	1 (0.8)	2 (1.6)
Non-drinker	1798 (44.2)	2272 (55.8)		2143 (52.7)	116 (2.9)	13 (0.3)

^*^Significant *p* value after FDR correction

Further analysis showed a significant negative association of prevalent anxiety with smoking, drinking behaviors, housework, and trip activities in total and male participants; and with housework and trip activities in female participants, and a positive association with diabetes (compared to hypertension) in all participants and those aged 50 to 70 years (compared to those less than 50) in male participants. No significant association was observed for BMI and exercise activities (Supplement Figure).

### The association between PA and anxiety

We further evaluated the associations of type of PA with the risk of anxiety after adjusting for potential confounders (Table 3). The housework and trip activities were inversely associated with the anxiety risk, with OR and 95%CI being 0.53 [0.45, 0.63] (*p* < 0.001) and 0.55[0.48, 0.63] (*p* < 0.001), respectively. We did not observe a significant association between exercise and the anxiety risk (OR 1.06, 95%CI: [0.94, 1.20], *p* = 0.321).

**Table 3:** Associations between physical activity and anxiety risk during COVID-19 lockdown

	No. of subjects	Model 1		Model 2		Model 3	
		OR (95% CI)	*P* value	OR (95% CI)	*P* value	OR (95% CI)	*P* value
Types of PA^a^							
Housework	3938	0.52 (0.45, 0.61)	< 0.001	0.53 (0.45, 0.63)	< 0.001	0.53 (0.45, 0.63)	< 0.001
Exercise	1998	1.06 (0.94, 1.20)	0.317	1.07 (0.95, 1.21)	0.289	1.06 (0.94, 1.20)	0.321
Trip activity	1461	0.56 (0.49, 0.63)	< 0.001	0.55 (0.48, 0.63)	< 0.001	0.55 (0.48, 0.63)	< 0.001
Levels of PA							
Low	814	Reference	/	Reference	/	Reference	/
Moderate	1797	0.52 (0.43, 0.62)	< 0.001	0.53 (0.44, 0.64)	< 0.001	0.53 (0.44, 0.64)	< 0.001
High	2210	0.50 (0.42, 0.59)	< 0.001	0.51 (0.42, 0.60)	< 0.001	0.51 (0.43, 0.51)	< 0.001

Model 1 adjusted for sex and age; model 2 additionally adjusted for BMI, smoking, and alcohol drinking; and model 3 additionally adjusted for BMI, smoking, alcohol drinking, and history of diseases

^a^OR (95%CI) for ever versus never of each type of PA

**Fig. 2:**
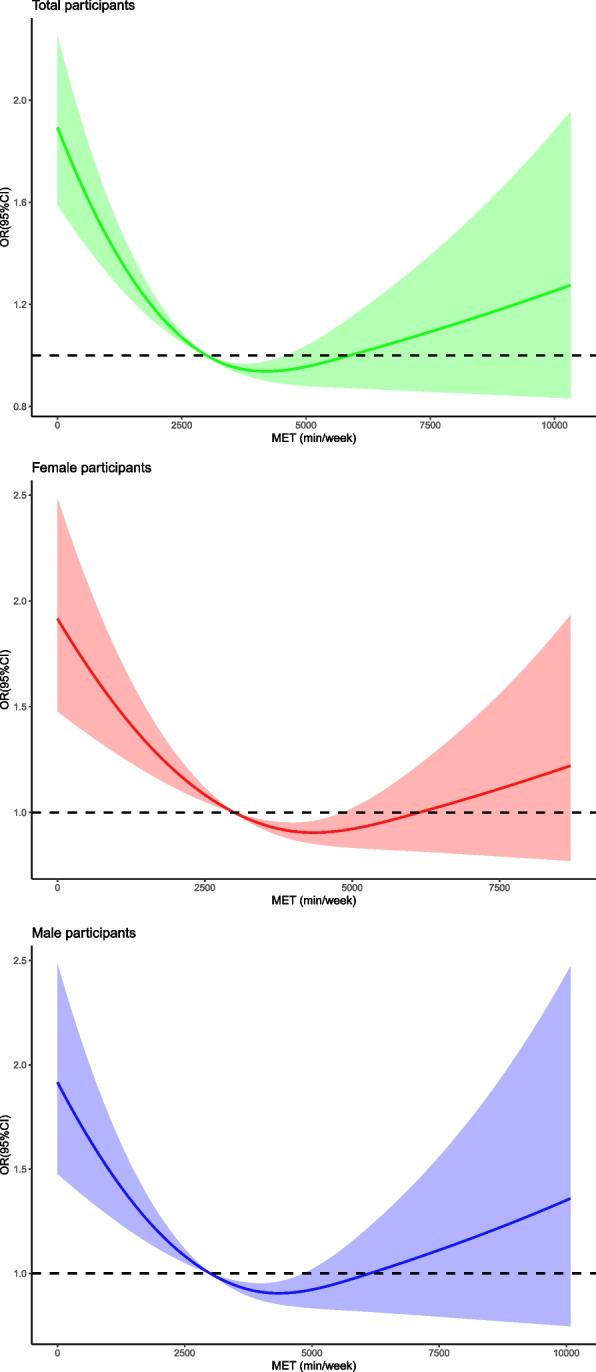
Association of anxiety symptom with METs in all participants (Figure 2.1), female (Figure 2.2) and male subjects s (Figure 2.3). The reference of METs for these plots (with OR fixed as 1.0) was 3360 min/week

We also explored the association between PA level and prevalent mild to severe anxiety among 4821 patients with complete data (Fig. 2 and Table 3). RCS curves demonstrated a significant non-linear relationship between PA level (METs) and the risk of anxiety symptom in all, female and male subjects (all p for non-linearity < 0.05). After adjusting for potential confounders, the subjects with moderate (OR 0.53, 95%CI: [0.44, 0.64], *p* < 0.001) or high PA level (OR 0.51, 95%CI: [0.43, 0.51], *p* < 0.001) had a lower prevalence of anxiety than those with low-level of PA. No significant difference was found between moderate and high PA level groups. The results of the short-scale were proved by using the long-scale (Supplement Table).

## Discussion

In this cross-sectional study, we found that more than half of patients suffered from anxiety (mostly mild). The prevalence of anxiety was higher in the females, the elders, non-smokers, non-drinkers, and patients with diabetes. A lower prevalence of anxiety was observed in patients having housework or trip activities, but no significant association was found in patients having exercise. Compared with patients with a low PA level, those with moderate or high PA levels had a lower risk of anxiety.

The 53.4% of anxiety prevalence in our subjects was higher than 5.3% in the general population [33], 35% in people suffering from COVID-19 [34], and 38.9% in diabetes patients before the outbreak of COVID-19 [35]. There are several explanations for the high prevalence of anxiety in our subjects. First, metabolic disorders have been associated with increased risk of psychological problems [35]. The susceptibility of diabetes patients to mental distress may contribute to the higher prevalence of anxiety in the population. Second, hypertension and diabetes were common comorbidities of COVID-19 in China and were considered adverse factors for a worse prognosis of COVID-19 [36, 37]. Patients under these chronic conditions might recognize their susceptibility to COVID-19 and thus be more worried about the risk [38]. However, we could not attribute the higher anxiety level in our subjects to any specific sources. It is note that most patients had a GAD-7 score of 5 to 9 and suffered mild anxiety, partly releasing the concern on the possible influence of severe events like losing loved ones due to COVID-19, and the worsening of prevalent diseases.

Previous results were inconsistent regarding the relationship of age and sex with mental health during the COVID-19 Pandemic Lockdown. Ding, et al. [1] found that anxiety and depression symptoms among women and young adults were aggravated during the COVID-19 pandemic lockdown. However, an opposite result was observed in another study, in which a higher risk of mental health symptoms was observed among men and younger participants [5]. In this study, we did not find a significant difference in prevalence of anxiety by sex and age groups. The discrepancy may be due to different risk exposures across populations and by sex and age, and is warranted further investigation.

The negative associations of smoking and drinking with anxiety may be attributed to the released mental distress by the behaviors. However, previous studies indicated improving or worsening mental status due to substance abuse [39–41]. It is also possible that psychological problems might urge patients to restrict themselves. Several studies found that the pandemic increased people’s substance use due to social isolation and/or psychological problems [40, 42]. Louvardi et al. [43] indicated that patients with respiratory diseases worried more during the pandemic, and reduced substance use for fear of COVID-19 and related respiratory diseases and comorbidities. In our subjects, the self-reported average daily cigarettes consumption dropped from 16 (before lockdown) to 12 (during lockdown), supporting the impact of anxiety on behaviors. We also found that there were more patients (1051 cases) reporting abnormal fasting glucose than those (573 cases) reporting abnormal systolic blood pressure (Supplement Note). The more concerns on medications deficiency and medical visits in diabetes patients compared with those having hypertension only may help to explain the higher anxiety level in diabetes patients.

Lockdown policy has largely changed people’s lifestyles, including PA type and PA level. Housework becomes a feasible subtype of PA during the pandemic, and was found to extricate people from the pressure of COVID-19 and improve their mental health [44]. In this study, we observed a significant association of housework with a reduced risk of anxiety, consistent with Asztalos, et al.’s results [44]. We also found a protective effect of trip activities (biking or walking) on anxiety, suggesting the effectiveness of outdoor activities in releasing psychological problems and improving mental health status. Under the lockdown situation, however, outdoor activities were greatly restricted. Therefore, the American College of Sports Medicine and WHO recommended 150 min of moderate home-based exercise per week during the lockdown [45, 46]. However, we did not observe a significant association between home-based exercise and the anxiety risk, which was inconsistent with other studies [47, 48]. This may be due to limited duration and intensity of home-based exercises in patients during the lockdown.

Regarding the PA level, previous studies indicated that the lockdown has greatly altered the duration and intensity of PA in general population. The decreased PA was a significant predictor for more severe insomnia symptom and deteriorated mental well-being, while more PA was associated with better well-being, improved anxiety, and sleep quality during the COVID-19 restriction [49–51]. Several studies suggested the protective effect of PA level on anxiety symptoms during the pandemic [52, 53]. A similar trend was also found for depression symptoms during the lockdown period [54]. Consistent with these previous studies, we observed a nonlinear relationship between PA level in METs and the risk of anxiety. The groups with moderate or high level of PA had a lower prevalence of anxiety than the group having a low PA level. The inverse association remained significant after adjusting for sex, type of chronic diseases, and other factors. Our findings suggest that the moderate, home-based PA may alleviate the anxiety symptoms among patients with non-communicable diseases during the COVID-19 lockdown period.

To the best of our knowledge, this is the first study systematically investigating the prevalence of and PA level associated with anxiety symptoms among non-communicable diseases patients during the COVID-19 lockdown period in China. The strengths of this study include the large sample size of non-communicable diseases patients, the rigorous multi-stages random sampling, the well-trained interviewers with medical background (medical students and community healthcare professionals), the detailed information collected and intensive analysis of the data, particularly the usage of the validated scales (IPAQ and GAD-7) to assess the anxiety and PA levels. All of which guarantee the high quality of data accurate estimations of the prevalence, and valid evaluation of the associations.

Our study had several limitations. First, as the cross-sectional design of this study, we could not confirm the causal relationship between PA and psychological symptoms. However, considering that PA is a lifestyle behavior that could not change substantially, we could make causal inference in a certain degree; for demographic and other factors, we could infer their protective or adverse effects on anxiety risk according to the chronological order. Second, the data was collected through phone-call interview in this study. Some events such as losing loved ones during the COVID-19 epidemic or severity of prevalent diseases might influence their responses to the questions asked by telephone. The non-negligible subjectivity and heterogeneity in both interviewers and subjects may have led to information bias. To minimize the potential bias, we required the interviewers to collect information on PA and anxiety using well-developed scales like IPAQ and GAD-7, both of which have been validated and proved effective in Chinese population. Finally, we did not collect any information on reasons for anxiety in this study, and thus could not attribute the high risk of anxiety in our subjects to any specific factors.

In summary, we found a higher prevalence of anxiety in patients with hypertension, diabetes, or both during the COVID-19 lockdown, and observed an inverse association of moderate PA with the risk of anxiety, particularly among the elders and diabetes patients. Our results highlight the potential benefit of housework and trip activities in mitigating mild to severe anxiety among this vulnerable population, and provide evidence for developing an effective intervention to improve their mental health in the context of the ongoing epidemic of COVID-19.

## Supplementary Information

**Additional file 1.**

## Data Availability

Given the sensitivity of the dataset, any researchers who intend to use the dataset could contact corresponding authors to get access.

## Abbreviations

PA: Physical activities
IPAQ: International Physical Activity Questionnaire
GAD-7: Generalized Anxiety Disorder 7-item
MET: Metabolic Equivalent for Task
