# Supplementary material for: Anxiety prevalence and its association with physical activity in patients with non-communicable diseases during COVID-19 lockdown: a cross-sectional study in Shanghai, China
Source: BMC Public Health. 2023 Feb 13;23:317. doi: 10.1186/s12889-022-14369-1 (PMC9924212; doi:10.1186/s12889-022-14369-1)
Supplement: Supplementary file 1 — Additional file 1. [file 12889_2022_14369_MOESM1_ESM.docx]

Supplementary Material

# Supplement Note 1: Basic information and characteristics of the investigation.

**Patients selection**

1) We randomly selected communities from 20 urban areas (20 of 116 communities), 10 urban-rural junction areas (10 of 45 communities), and 10 rural areas (10 of 58 communities) of Shanghai. We then randomly selected 200 patients with hypertension or diabetes in each chosen community by simple random sampling (considering 25% of non-response rate).

2) A total of 8,000 survey subjects with hypertension or diabetes or both were selected, and 7705 individuals were able to be contacted with valid phone number. After the phone call survey, we received 4994 questionnaires.

3) Since this investigation was a telephone survey, we made questionnaires inclusion criteria:

(1) The date of recorded telephone survey was from Feb. 25 2020 to Apr. 20 2020; questionnaires were excluded if recorded date was not within this period, or no recorded date, or invalid information (for example, not a date format) in the recorded date site.

(2) Patients with self-reported clear information of hypertension and/or diabetes; questionnaires were excluded if patients replied that they did not had either of the two diseases, or no information, or invalid information in the recorded date site.

(3) Validified IPAQ and GAD-7 score; questionnaires were excluded if the score site was non-numeric information.

Questionnaires not matched with these 3 requirements were excluded. We finally had 4877 validified questionnaire responses.

In addition to the basic characteristics showed in table 1, we also included: “the amount of cigarettes before lockdown” (median 15, IQR: [10, 20], NA: 3552), “the amount of cigarettes during lockdown” (median 10, IQR: [5, 20], NA: 3661), “the systolic blood pressure measured last time” (normal: 3641, lower: 22, higher: 559, unknown: 650, NA: 5 ), “the fasting blood sugar measured last time” (normal: 2467, lower: 10, higher: 1034, unknown: 1366, NA: 0 ).

Given the controversy of IPAQ answers, we tidied up the dataset and clear the data followed with Chinese version IPAQ standards (described in the main text), and finally we had 4821 participants in RCS analyses.

# Supplement Note 2: Questionnaire on self-management behavior and anxiety status of community patients with chronic diseases during COVID-19 lock-down period.

**Dear Participants：**

The purpose of this survey is to investigate the changes of self-management behaviors of chronic diseases such as smoking, drinking, diet and exercise of patients with hypertension and diabetes in the community before and after the COVID-19 lock-down period, and the anxiety prevalence of patients with hypertension and diabetes in the community and its influencing factors during the COVID-19 lock-down period, so as to provide a scientific basis for optimizing and improving the health management strategy of chronic diseases in the community. This study was funded by Fudan University and Shanghai Municipal Center for Disease Control and Prevention, and implemented by Shanghai Municipal Center for Disease Control and Prevention. We sincerely invite you to participate in this study. All information collected in this study, including demographic information, smoking, drinking, diet, physical activity, medical therapy and anxiety status, is strictly confidential. All members of this research team will sign a confidentiality agreement. Whether you participate in this survey is entirely voluntary. You can refuse to participate, or you can quit at any time during the survey. If you refuse to participate or quit, it will not affect any of your interests. This study was approved by the Institutional Review Board of the Fudan University School of Public Health.

**Part 1 Demographic information**

| A1 Name: A2 Sex： 1… Male 2… Female  A3 ID: A4 Birthday:  A5 Diabetes mellitus：1… Yes 2 … No A6 Hypertension: 1… Yes 2… No  A7 Height: cm A8 Weight: kg A9 Waist: cm  A10 Investigator： A11 Survey date： |
| --- |

**Part 2 Physical activity**

| **Think about only those physical activities that you did for at least 10 minutes at a time.** | **Before the epidemic of COVID-19** | **COVID-19 lock-down** |
| --- | --- | --- |
| **B Exercise activity** | | |
| B1.1 During the last 7 days, did you walk for at least 10 minutes at a time in your leisure time? (No walking in leisure time Skip to question B2.1) | 1… Yes 2… No | 1… Yes 2… No |
| B1.2 How many days did you walk for at least 10 minutes at a time in your leisure time? | Days per week | Days per week |
| B1.3 How much time did you usually spend on one of those days walking in your leisure time? | Hours per day  Minutes per day | Hours per day  Minutes per day |
| B2.1 During the last 7 days, did you do moderate physical activities like bicycling at a regular pace, swimming at a regular pace, and doubles tennis in your leisure time? (No moderate physical activities in leisure time Skip to question B3.1) | 1… Yes 2… No | 1… Yes 2… No |
| B2.2 How many days did you do moderate physical activities like bicycling at a regular pace, swimming at a regular pace, and doubles tennis in your leisure time? | Days per week | Days per week |
| B2.3 How much time did you usually spend on one of those days doing moderate physical activities in your leisure time? | Hours per day  Minutes per day | Hours per day  Minutes per day |
| B3.1 During the last 7 days, did you do vigorous physical activities like aerobics, running, fast bicycling, or fast swimming in your leisure time? (No vigorous activity in leisure time Skip to question C1.1) | 1… Yes 2… No | 1… Yes 2… No |
| B3.2 How many days did you do vigorous physical activities like aerobics, running, fast bicycling, or fast swimming in your leisure time? | Days per week | Days per week |
| B3.3 How much time did you usually spend on one of those days doing vigorous physical activities in your leisure time? | Hours per day  Minutes per day | Hours per day  Minutes per day |
| **C Homework activity** | | |
| C1.1 During the last 7 days, did you do vigorous physical activities like heavy lifting, chopping wood, shoveling snow, or digging in the garden or yard? (No vigorous activity in garden or yard Skip to question C2.1) | 1… Yes 2… No | 1… Yes 2… No |
| C1.2 How many days did you do vigorous physical activities like heavy lifting, chopping wood, shoveling snow, or digging in the garden or yard? | Days per week | Days per week |
| C1.3 How much time did you usually spend on one of those days doing vigorous physical activities in the garden or yard? | Hours per day  Minutes per day | Hours per day  Minutes per day |
| C2.1 During the last 7 days, did you do moderate activities like carrying light loads, sweeping, washing windows, and raking in the garden or yard? (No moderate activity in garden or yard Skip to question C3.1) | 1… Yes 2… No | 1… Yes 2… No |
| C2.2 How many days did you do moderate activities like carrying light loads, sweeping, washing windows, and raking in the garden or yard? | Days per week | Days per week |
| C2.3 How much time did you usually spend on one of those days doing moderate physical activities in the garden or yard? | Hours per day  Minutes per day | Hours per day  Minutes per day |
| C3.1 During the last 7 days, did you do moderate activities like carrying light loads, washing windows, scrubbing floors and sweeping inside your home? (No moderate activity inside home Skip to C4.1) | 1… Yes 2… No | 1… Yes 2… No |
| C3.2 How many days did you do moderate activities like carrying light loads, washing windows, scrubbing floors and sweeping inside your home? | Days per week | Days per week |
| C3.3 How much time did you usually spend on one of those days doing moderate physical activities inside your home? | Hours per day  Minutes per day | Hours per day  Minutes per day |
| **D Trip activity** | | |
| D1.1 During the last 7 days, did you travel in a motor vehicle like a train, bus, car, or tram? (No traveling in a motor vehicle Skip to question D2.1) | 1… Yes 2… No | 1… Yes 2… No |
| D1.2 How many days did you travel in a motor vehicle like a train, bus, car, or tram? | Days per week | Days per week |
| D1.3 How much time did you usually spend on one of those days traveling in a train, bus, car, tram, or other kind of motor vehicle? | Hours per day  Minutes per day | Hours per day  Minutes per day |
| D2.1 During the last 7 days, did you bicycle for at least 10 minutes at a time to go from place to place? (No bicycling from place to place Skip to question D3.1) | 1… Yes 2… No | 1… Yes 2… No |
| D2.2 How many days did you bicycle for at least 10 minutes at a time to go from place to place? | Days per week | Days per week |
| D2.3 How much time did you usually spend on one of those days to bicycle from place to place? | Hours per day  Minutes per day | Hours per day  Minutes per day |
| D3.1 During the last 7 days, did you walk for at least 10 minutes at a time to go from place to place?(No walking from place to place Skip to E1.1) | 1… Yes 2… No | 1… Yes 2… No |
| D3.2 How many days did you walk for at least 10 minutes at a time to go from place to place? | Days per week | Days per week |
| D3.3 How much time did you usually spend on one of those days walking from place to place? | Hours per day  Minutes per day | Hours per day  Minutes per day |
| **E Sedentary activity** | | |
| E1.1During the last 7 days, how much time did you usually spend sitting on a weekday? | Days per week | Days per week |
| E1.2 During the last 7 days, how much time did you usually spend sitting on a weekend day? | Hours per day  Minutes per day | Hours per day  Minutes per day |

**Part 3 Dietary, Smoking and Drinking**

F1 How about your diet control during the COVID-19 lock-down period?

1…Follow the doctor's advice 2…Not control 3…Irregular

F2 Did you eat more or less than usual during the COVID-19 lock-down period?

1…Significant increase 2…Slightly increase 3…Significant reduce

4…Slightly reduce 5…No change

F3 How much did you eat the following foods during the COVID-19 lock-down period?

| Food | Intake  （g/day） | Compared with your general eating habits, how about your intake of the following foods during the COVID-19 lock-down period? | | | | |
| --- | --- | --- | --- | --- | --- | --- |
|  |  | Significant increase | Slightly increase | Significant reduce | Slightly reduce | No change |
| F3.1 Staple food (Rice and noodles) |  | 1 | 2 | 3 | 4 | 5 |
| F3.2 Meats (Pigs,cattle, sheep, chickens and ducks) |  | 1 | 2 | 3 | 4 | 5 |
| F3.3 Fish, shrimp and crab |  | 1 | 2 | 3 | 4 | 5 |
| F3.4 Eggs |  | 1 | 2 | 3 | 4 | 5 |
| F3.5 Milk（ml/day） |  | 1 | 2 | 3 | 4 | 5 |
| F3.6 Vegetables |  | 1 | 2 | 3 | 4 | 5 |
| F3.7 Fruits |  | 1 | 2 | 3 | 4 | 5 |
| F3.8 Tofu and soybean products |  | 1 | 2 | 3 | 4 | 5 |
| F3.9 Mushrooms |  | 1 | 2 | 3 | 4 | 5 |

F4.1 Have you smoked every day in the past month?

1… Yes 2… No (Skip to F5.1)

F4.2 If so, how many cigarettes have you smoked every day in the past month? per day

F5.1 Have you drank every day in the past month?

1… Yes 2… No (Skip to Part4)

F5.2 If so, how much alcohol did you drink every day during the past month? ______ml

F5.3 If so, what kind of wine do you mainly drink?

1… Rice wine 2… Beer 3… White spirit 4… Wine

**Part 4 Medication**

G1 Did you take hypoglycemic or anti-hypertensive drugs for a long time?

1… Yes 2… No ( Skip to Part5)

G2 How many kinds of hypoglycemic and anti-hypertensive drugs did you take for a long time?

1… 1 2… 2-3 3… 3-5 4… ≥5

G3 What kind of hypoglycemic and anti-hypertensive drugs did you take?

| Drug name | Drug dose | Drug dose unit  (1…Tablet (Grain) 2…mg 3…ml) | Frequency  （Times /per day） |
| --- | --- | --- | --- |
|  |  |  |  |
|  |  |  |  |
|  |  |  |  |
|  |  |  |  |

G4 During the COVID-19 lock-down period, how did you get hypoglycemic and anti-hypertensive drugs?

1…Hospital 2…Pharmacy 3…Internet 4…Others, please list____ __

G5 During the COVID-19 lock-down period, did you take medicine according to the doctor's advice?

1…Follow the doctor's advice 2…Irregular and sometimes missing

G6 During the COVID-19 lock-down period, did you stop taking drugs or switch to other drugs because you could not obtain drugs for long-term use?

1… Yes 2… No ( Skip to I1)

G7 If yes, please listed the name of the drug not obtained in time, the days of withdrawal and the name of alternative drugs.

| Drug name | Days | Are alternative drugs used? | If yes, which alternative drugs are used? |
| --- | --- | --- | --- |
|  |  | 1… Yes 2… No |  |
|  |  | 1… Yes 2… No |  |
|  |  | 1… Yes 2… No |  |
|  |  | 1… Yes 2… No |  |
|  |  | 1… Yes 2… No |  |

G8 During the COVID-19 lock-down period, did you need to see a doctor because of hypertension, diabetes or other chronic diseases?

1… Yes, need to go to he comprehensive hospital 2 … Yes, need to go to the CHCS 3…No

**Part 5 Generalized Anxiety Disorder 7-item (GAD-7) scale**

Over the last two weeks, how often have you been bothered by the following problems?

|  | Not at all | Several days | More than half the days | Nearly every day |
| --- | --- | --- | --- | --- |
| H1 Feeling nervous, anxious, or on edge (nervousness) |  |  |  |  |
| H2 Not being able to stop or control worrying (inability to stop worrying) |  |  |  |  |
| H3 Worrying too much about different things (excessive worry) |  |  |  |  |
| H4 Trouble relaxing (restlessness) |  |  |  |  |
| H5 Being so restless that it is hard to sit still (difficulty in relaxing) |  |  |  |  |
| H6 Becoming easily annoyed or irritable (easy irritation) |  |  |  |  |
| H7 Feeling afraid, as if something awful might happen (fear of something awful happening) |  |  |  |  |

# Supplement
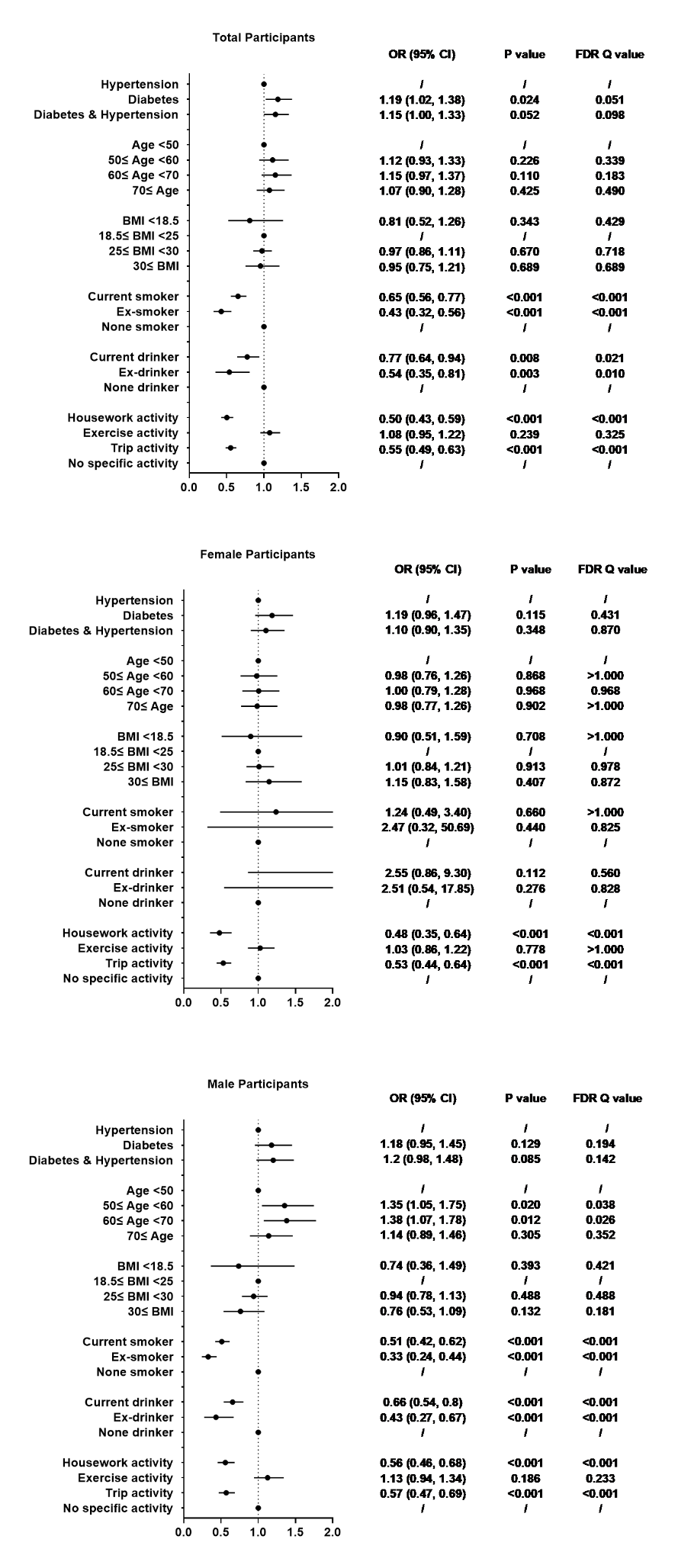
 figure 1：The association of different factors with the risk of anxiety.

# Supplementary Table: Comparing the results of long-scale and short-scale of IPAQ.

|  | Category | No. | Model 1 | | Model 2 | | Model 3 | |
| --- | --- | --- | --- | --- | --- | --- | --- | --- |
|  |  |  | OR (95% CI) | P value | OR (95% CI) | P value | OR (95% CI) | P value |
| Short IPAQ | Low PA | 814 | Reference | / | Reference | / | Reference | / |
|  | Moderate PA | 1797 | 0.52 (0.43, 0.62) | <0.001 | 0.53 (0.44, 0.64) | <0.001 | 0.53 (0.44, 0.64) | <0.001 |
|  | Vigorous PA | 2210 | 0.50 (0.42, 0.59) | <0.001 | 0.51 (0.42, 0.60) | <0.001 | 0.51 (0.43, 0.51) | <0.001 |
| Long IPAQ | Low PA | 828 | Reference | / | Reference | / | Reference | / |
|  | Moderate PA | 2021 | 0.55 (0.46, 0.65) | <0.001 | 0.55 (0.46, 0.66) | <0.001 | 0.55 (0.46, 0.66) | <0.001 |
|  | Vigorous PA | 1972 | 0.48 (0.40, 0.57) | <0.001 | 0.48 (0.40, 0.58) | <0.001 | 0.48 (0.40, 0.58) | <0.001 |
